# Robotic Partial Nephrectomy with the Da Vinci Xi

**DOI:** 10.1155/2016/9675095

**Published:** 2016-02-09

**Authors:** George J. S. Kallingal, Sanjaya Swain, Fadi Darwiche, Sanoj Punnen, Murugesan Manoharan, Mark L. Gonzalgo, Dipen J. Parekh

**Affiliations:** Department of Urology, University of Miami Miller School of Medicine, Miami, FL 33136, USA

## Abstract

*Purpose.* The surgical expertise to perform robotic partial nephrectomy is heavily dependent on technology. The Da Vinci Xi (XI) is the latest robotic surgical platform with significant advancements compared to its predecessor. We describe our operative technique and experience with the XI system for robotic partial nephrectomy (RPN).* Materials and Methods.* Patients with clinical T1 renal masses were offered RPN with the XI. We used laser targeting, autopositioning, and a novel “in-line” port placement to perform RPN.* Results.* 15 patients underwent RPN with the XI. There were no intraoperative complications and no operative conversions. Mean console time was 101.3 minutes (range 44–176 minutes). Mean ischemia time was 17.5 minutes and estimated blood loss was 120 mLs. 12 of 15 patients had renal cell carcinoma. Two patients had oncocytoma and one had benign cystic disease. All patients had negative surgical margins and pathologic T1 disease. Two postoperative complications were encountered, including one patient who developed a pseudoaneurysm and one readmitted for presumed urinary tract infection.* Conclusions.* RPN with the XI system can be safely performed. Combining our surgical technique with the technological advancements on the XI offers patients acceptable pathologic and perioperative outcomes.

## 1. Introduction

Since the new millennium, robotic surgery has been increasingly utilized for a variety of procedures including robotic partial nephrectomy (RPN). Compared to laparoscopic partial nephrectomy, RPN is technically less difficult and is associated with less chance of conversion to radical nephrectomy [1], less blood loss [2–4], shorter ischemia times [2, 5], and shorter hospital stay [2, 3]. The robotic platform allows better articulation of the wrists and improved vision in 3 dimensions, which has facilitated a shorter learning curve for surgeons adopting minimally invasive surgery. Since the year 2000, when the Da Vinci robot was first utilized, the robot has undergone several iterations, each with tremendous technological advancement over the prior version. Recently, Intuitive Surgical has introduced their latest upgrade to the robot, the Da Vinci Xi (Intuitive Surgical, Sunnyvale, CA). In this paper we detail our first experiences and lessons learned with the Da Vinci Xi system for RPN.

## 2. Materials and Methods

Beginning May 2014, patients who were found to have clinical stage 1 renal masses were offered surgery, surveillance, or ablative therapy, in concordance with the AUA 2009 guidelines for the clinical stage 1 renal mass [6]. If amenable to partial nephrectomy, patients were offered RPN with the Da Vinci Xi surgical system. Patients with T2 masses or highly complex renal masses where partial nephrectomy was not deemed feasible were not included in this study. Patients had a full preoperative evaluation prior to surgery. Routine laboratory evaluation, including serum creatinine, was performed in all patients. The surgeon evaluated the preoperative CT scan and MRI and graded complexity using the RENAL Nephrometry scoring system [7]. All patients were given written informed consent prior to surgery. This study met IRB approval.

### 2.1. General Considerations

**Figure 1:**
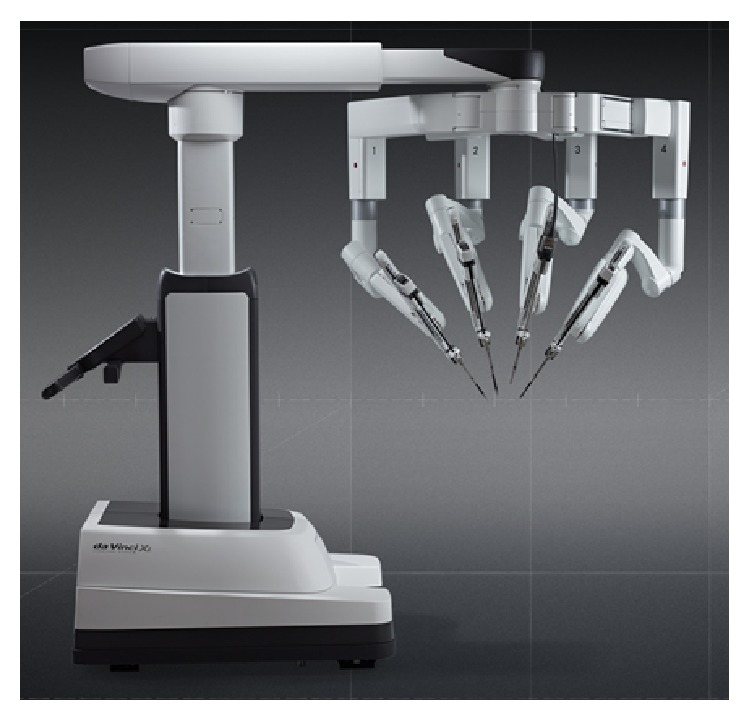
Da Vinci Xi patient cart.

The Da Vinci Xi Surgical Platform (Figure 1) includes numerous technological enhancements. In particular, significant improvements center on the patient cart and docking process. The patient cart features four robotic arms mounted on movable overhead boom, which allows 342 degrees of rotation and docking from any quadrant. Laser crosshairs on the boom facilitate aligning the patient cart with the designated camera port. The robotic arms are thinner and have additional joints (patient clearance joints) that allow rotation away from the patient. The endoscope has been redesigned to 8 mm and can be placed into any working robotic port, allowing for camera port hopping. After the trocars have been inserted, the camera port is docked first and the camera is focused on the target anatomy. The autotargeting feature then allows the remaining robotic arms to autorotate on the boom to minimize clashing and optimize performance.

### 2.2. Technique

#### 2.2.1. Patient Positioning

**Figure 2:**
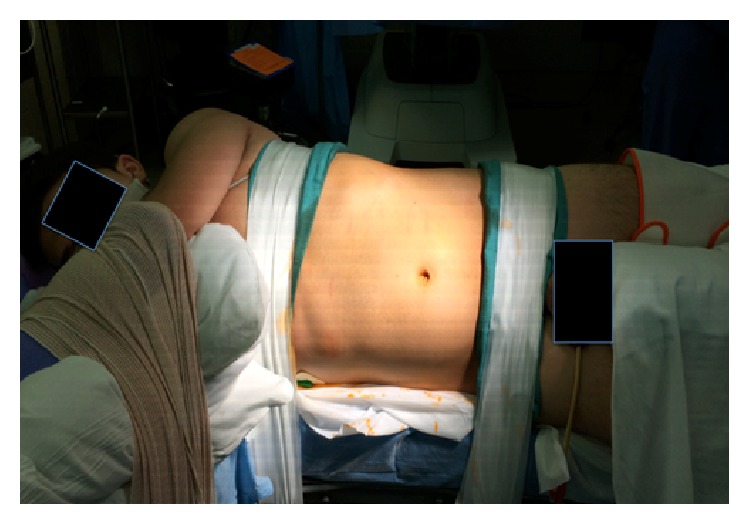
Patient positioning for left robotic partial nephrectomy.

A Foley catheter and nasogastric tube are placed prior to positioning. The patient is then placed in a modified lateral decubitus position at 45 degrees with a gel roll supporting the lower back. The anterior abdomen is placed on the lateral edge of the bed to minimize interference with the operative table. All pressure points are padded. An axillary role is placed and the upper arm is secured over the torso with pillows (see Figure 2). Heavy silk tape is applied over the chest and hips to secure the patient to the operative bed. A pliable wrap is used to secure the arms in place.

#### 2.2.2. Port Placement

**Figure 3:**
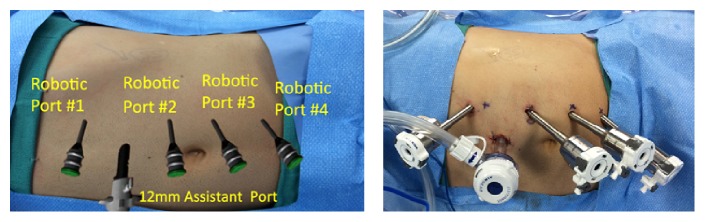
Port placement for left robotic partial nephrectomy.

The assistant port is placed first using an open Hasson technique. We use an Airseal (SurgiQuest Inc., Milford, CT) port placed in the midline, three to four centimeters above the umbilicus. After pneumoperitoneum is achieved, the laparoscope is inserted and the peritoneal cavity is inspected for injuries or adhesions. Four robotic ports are placed under direct vision in a linear fashion at the lateral border of the rectus muscle (Figure 3). These ports are spaced about 6 cm apart. Typically, the second most cephalad port (port #2) is intended for the camera and should be below the level of the renal hilum. We also ensure there is enough space between port #1 and port #2, to accommodate the assistant port. For right-sided procedures, we place a 5 mm subxiphoid port for liver retraction. Ports for a left RPN are shown in place in Figure 3.

#### 2.2.3. Docking

**Figure 4:**
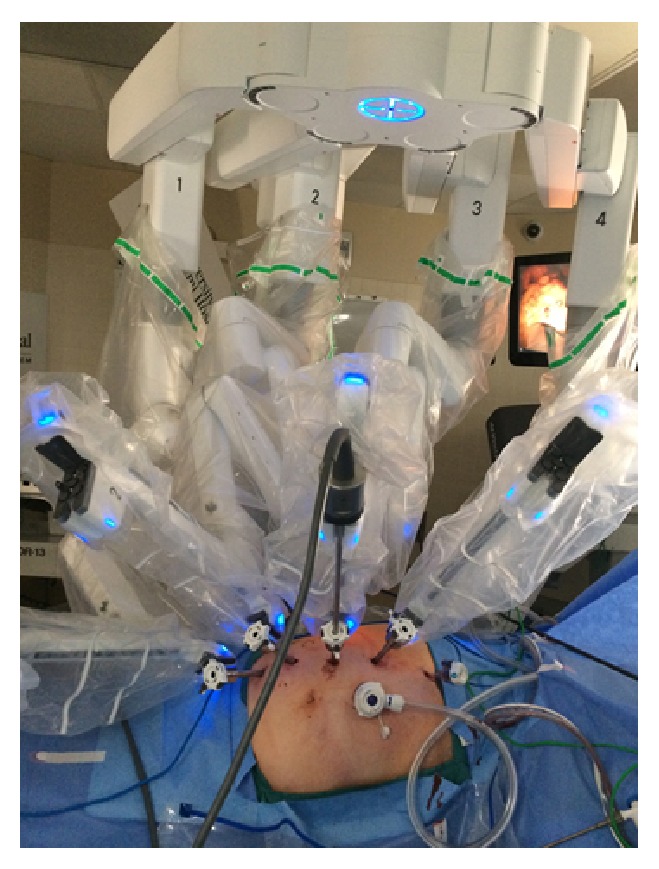
XI robot docked for right robotic partial nephrectomy.

When the patient cart is driven for docking, the laser guidance is activated to facilitate precise positioning. The laser crosshairs projected from the overhead boom are aligned with the designated camera port. The camera port (port #2) is then mounted to the robotic arm and the camera is inserted. We focus the camera on the anticipated location of the renal hilum and then activate the autotargeting feature. Since the renal hilum is not visible on initial port placement, we have used external cues (subcostal region) in addition to internal cues (posterior to lower liver on the right side, or several inches caudad to the spleen on the left). The autotargeting allows for optimal boom rotation and robotic arm placement, to maximize access and minimize collision. After autopositioning, the remaining cannulas are docked and the robotic instruments are placed. The robotic arms are moved close together to minimize clashing (Figure 4). Lastly, the patient clearance joints on arms #1 and #4 are rotated toward the patient to maximize arm movement.

#### 2.2.4. Robotic Partial Nephrectomy

The camera is routinely placed in port #2. The remaining robotic instruments include a fenestrated bipolar, monopolar scissors, and a ProGrasp in ports 1, 3, and 4, respectively. In order to expose Gerota's fascia, we divide the white line of Toldt and retract the colon medially. The inferior aspect of Gerota's is developed until the ureter is identified. The ProGrasp in the 4th arm is used to elevate the ureter off the psoas muscle, allowing a clear path to the renal hilum. Next, we expose the renal vessels. All major arterial branches are identified and mobilized from surrounding structures. We then focus on identifying our tumor. The perirenal fat is gently elevated from the kidney capsule to create wide exposure. For posteriorly based tumors, we will separate the perirenal fat around the entire kidney, in order to reflect the kidney 180 degrees. Once the tumor is identified, we introduce an ultrasound probe to identify the depth and margins of the tumor. Using the cautery, the margins of the tumor are scored. For hilar control, Reliance bulldog clamps (Scanlan International, St. Paul, MN) are introduced laparoscopically and passed to the ProGrasp. All major arterial branches are clamped with the Reliance bulldog clamps initiating renal ischemia. The 4th arm is used to keep the kidney rotated in the optimal position. The scissors are used to cut the tumor without cautery, while the fenestrated bipolar is used to retract the tumor. Liberal use of laparoscopic suction is critical in maintaining excellent vision and ensuring benign-appearing tumor margins. Once the tumor is completely excised, it is placed above the liver or spleen. A biopsy of the deep margin of the tumor base and any suspicious areas are then taken and passed off immediately, as per custom institutional practice. The left and right robotic instruments are then changed to large robotic needle drivers. Any discrete bleeding vessels or defects in the collecting system are closed with a 2-0 barbed suture. The kidney defect is then closed in two layers using the sliding renorrhaphy technique with barbed suture and hem-o-lok clips [8]. Once hemostasis is assured, the bulldog clamps are released from the renal vessels. The renal closure and hilum are inspected and the tumor is placed in an endocatch bag and extracted. We then place a drain in the lateral renal fossa. Closing procedures are then performed in standard fashion.

## 3. Results

From May to July 2014, 15 patients with clinical stage 1 renal masses underwent RPN with the Da Vinci Xi surgical system. RPN was able to be performed successfully in all patients without conversion to radical nephrectomy or conversion to open partial nephrectomy.  Table 1 lists the demographic and preoperative patient characteristics. The mean patient age was 61 years and roughly half were women. The mean patient BMI was 30.6. The mean preoperative eGFR was 76.1 mL/min/1.73 m^2^. Five patients had chronic kidney disease stage 3 or higher. All patients had preoperative CT or MRI. The mean tumor size was 2.74 cm. The patients harbored a wide range of tumor complexity. The median RENAL complexity score was moderate. Seven patients had low complexity lesions and three patients had highly complex lesions.

For operative parameters (Table 2), the mean console time was 101.3 minutes and the mean port placement time was 17.5 minutes. An average of 120 mLs of blood was lost during the case and the median hospital stay was 2 days. The ischemia time was consistently short with mean time of 17.5 minutes and range of 0–40 minutes. One patient was done off-clamp.

12 of 15 patients had renal cell carcinoma on final pathology (seven with clear cell, two with papillary type 1, and three with chromophobe). Three patients had benign disease with one having benign cystic disease and two patients with oncocytoma. All surgical margins were negative and all patients had pT1 disease. All deep base biopsies were negative as well. All patients were discharged by POD #3 and 66% were discharged by POD #2. Two perioperative complications were noted. One 30-year-old male patient had a drop in hemoglobin on the first postoperative day (POD) from 14 to 6 gm/dL. Angiogram showed a pseudoaneurysm in a small artery at the lower margin of the defect. This was embolized with success. He was transfused 2 units and discharged on POD #3. The second complication was a readmission for possible UTI in a forty-year-old woman on POD #4. Cultures and imaging were negative and she was discharged home.

## 4. Discussion

For the experienced robotic surgeon, the RPN could be performed seamlessly on the XI with several notable differences. Port placement is significantly different on the XI with this procedure compared to the Si. The XI is designed for parallel movement, which means the instruments work best when they are working in a near parallel configuration. This makes port placement ideal when the ports are in a line. In contrast, with the Si, the 4th arm port is typically placed lateral to the other ports. This lateral placement often would cause external or internal collision. For the RPN, the 4th arm port placement in the midline is advantageous and provides more functionality with minimal collision. On the XI, we are able to utilize the 4th arm for multiple tasks including bowel retraction, elevation of the lower pole of the kidney, and holding the kidney in position during the extirpative and reconstructive phases of the surgery.

In addition, the ports can (and should) be significantly closer together compared to the Si. There should be 6 cm between ports on the XI, compared to 8–10 cm for the Si. When the ports are closely spaced, the arms are allowed to work in parallel with each other. Furthermore, the camera is 8 mm on the XI, which allows it to be placed in any of the robotic ports. Camera port hopping is thus far an exclusive feature of the Da Vinci Xi; however, we have not found it necessary for an RPN.

Perhaps the most pronounced difference in the XI is in the docking phase.

The XI patient cart occupies a different physical space than the Si. One major benefit is the addition of the boom, which allows the robotic arms to extend and rotate, in order to facilitate docking from multiple angles. This allows the patient to console more flexibility in positioning within the operative theatre. One drawback of the boom is that it creates additional height to the patient cart. In our operating rooms, this means navigating ceiling attachments more carefully. We have docked the robot both from the feet (with 90-degree boom rotation) and from the patient's side (perpendicular to the operative table). The side docking offers the most reliable targeting in our experience, irrespective of the patient's height.

Most of the operative technique for an RPN on the XI is similar to that on the Si. We have noted several changes with camera. The XI video camera is autofocusing, which can save time. However, the fidelity of tissue color on the camera can fluctuate, especially when there is blood in the field, sometimes giving an orange hue. Also, the smoke from the cautery is more obtrusive than on the Si. We have used the valveless Airseal port with continuous smoke suction to address this limitation. As a distinct advantage, the instruments for the XI are 1.75 inches longer, which facilitates longer reach and has aided upper pole kidney mobilization for the RPN.

Our console time and ischemia time were relatively short, even in the face of moderately complex tumors. Our mean ischemia time of 17.5 minutes and estimated blood loss (EBL) compare favorably to other large series of robotic partial nephrectomy with prior Da Vinci models [9, 10]. Long et al. describe a mean warm ischemia time of 19.2 min and mean EBL of 260 mLs in 400 patients treated with prior models of the Da Vinci robotic system [1]. Moreover, a recent meta-analysis included 23 different studies with 1152 patients treated by RPN and found a range of mean WIT of 18–35.5 minutes, and the range of mean EBL of 93 mLs to 490 mLs [6]. Although comparative trials are lacking, our perioperative data suggest that robotic partial nephrectomy with the Da Vinci Xi is at least comparable to robotic partial nephrectomy with previous versions of the robot. In the current study, we focused on T1 renal masses; however larger masses that are amenable to partial nephrectomy can be considered in the future. Patients with significant renal insufficiency and complex renal masses were offered open partial nephrectomy.

Overall, surgeons felt that the XI offered significant improvements over the Si for the RPN. Docking was more precise and instrument clashing was minimal. In particular, the XI added usefulness to the 4th arm and generally made the case flow more smoothly. Due to technological changes in the robot, the port placement and docking procedure are markedly different than typical procedures with the previous versions of the robot. Console time and ischemia time were expectedly short. Perioperative outcomes and pathologic outcomes were similar to our prior experience with RPN.

## 5. Conclusions

RPN with the Da Vinci Xi system can be performed in a safe and reproducible fashion. This latest upgrade provides several advantages over the previous system that can facilitate a more efficient procedure but further studies are required to fully elucidate performance outcomes in multiple settings.

## Figures and Tables

**Table 1:** Demographic and preoperative characteristics.

Age (yrs)	Sex	BMI	Preop eGFR (mL/min/1.73 m^2^)	Side	Imaging size (cms)	RENAL score	RENAL complexity
61	F	31	117.27	Right	3.9	9	Moderate
65	F	23	88.33	Right	2.8	8	Moderate
76	F	32	91.87	Left	2.4	5	Low
30	M	26	67.19	Left	1.7	7	Moderate
66	M	23	94.00	Right	3.2	10	High
79	M	25	41.90	Right	1.6	4	Low
49	M	36	79.41	Left	1.8	6	Low
62	F	34	72.68	Left	5.4	10	High
44	F	39	106.54	Left	2.0	7	Moderate
63	F	32	34.28	Left	3.6	4	Low
84	M	27	80.39	Right	2.1	6	Low
49	M	28	71.90	Left	2.6	10	High
71	M	35	51.22	Left	3	4	Low
57	F	23	97.40	Right	1.9	9	Moderate
54	M	37	47.31	Left	3	4	Low

**Table 2:** Operative and pathologic outcomes.

Console time (min)	Ischemia time (min)	EBL (mls)	Intraop. comp.	Pathological subtype	Tumor size (cms)	T stage	Surgical margin
99	40	100	No	Clear cell	3.2	1a	Neg.
98	19	50	No	Oncocytoma	2.5	n/a	Neg.
132	14	50	No	Chromophobe	2	1a	Neg.
77	20	100	No	Clear cell	1.6	1a	Neg.
111	20	100	No	Chromophobe	3.5	1a	Neg.
67	0	50	No	Benign cyst	n/a	n/a	Neg.
85	11	50	No	Papillary type 1	1.7	1a	Neg.
123	27	200	No	Clear	5	1b	Neg.
73	9	50	No	Chromophobe	2	1a	Neg.
96	15	100	No	Clear cell	3	1a	Neg.
106	22	200	No	Oncocytoma	2.5	n/a	Neg.
182	18	150	No	Clear cell	2.5	1a	Neg.
51	10	400	No	Papillary type 1	2.8	1a	Neg.
44	18	100	No	Clear cell	2	1a	Neg.
176	20	100	No	Clear cell	2.5	1a	Neg.
